# The “cutting edge” of non-canonical RNA splicing

**DOI:** 10.3389/fmolb.2026.1719817

**Published:** 2026-04-23

**Authors:** Jiusi Guo

**Affiliations:** Department of Biology, Georgia State University, Atlanta, GA, United States

**Keywords:** alternative splicing, non-canonical splicing, non-canonical splice sites, microexons, recursive splicing, trans-splicing, spliceosome-independent splicing, circular RNA

## Abstract

Splicing, including alternative splicing, is a fundamental post-transcriptional mechanism in eukaryotes that generates functional proteins and transcript diversity. Canonical splicing follows well-defined rules, such as sufficient intron and exon lengths, specific splice junction orientations, consensus dinucleotides at donor and acceptor sites, and mediation by the spliceosome. However, certain splicing events deviate from these canonical rules. This review synthesizes multiple forms of non-canonical splicing within a framework that reflects their increasing deviation from canonical mechanisms, including non-canonical splice sites, non-canonical splicing in lncRNAs, microexons, recursive splicing, trans-splicing, and spliceosome-independent splicing. In addition, this review provides a critical analysis of the current state of research for each form of non-canonical splicing and outlines key directions for future investigation. As a case study, we reanalyzed RNA-seq data from mouse neuronal cells to further examine non-canonical splice sites. These analyses show that non-canonical introns tend to be shorter and that many non-canonical junctions retain at least one canonical donor or acceptor dinucleotide, supporting the view that a substantial subset remains compatible with spliceosome-mediated recognition. Together, this review provides a structured perspective on how canonical splicing rules can be relaxed, repurposed, or bypassed across distinct biological contexts.

## 1 Introduction to RNA splicing

**FIGURE 1:**
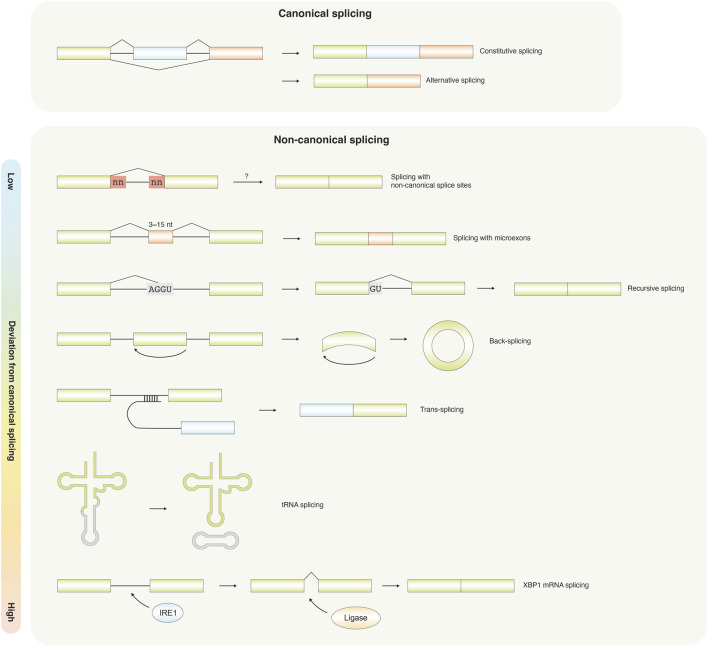
An overview flowchart illustrating canonical and non-canonical splicing. Canonical splicing comprises constitutive splicing and alternative splicing, whereas non-canonical splicing encompasses splicing with non-canonical splice sites, non-canonical splicing in long non-coding RNAs (lncRNAs), microexons, recursive splicing, trans-splicing, and spliceosome-independent splicing.

**FIGURE 2:**
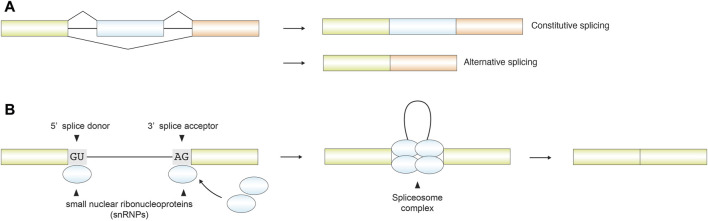
**(A)** Schematic representation of canonical splicing. In constitutive splicing, all exons within a pre-mRNA are sequentially ligated in their genomic order to generate a single mature transcript. In contrast, alternative splicing selectively includes or skips specific exons, producing multiple RNA isoforms that can be translated into distinct protein variants. **(B)** Simplified mechanism of canonical splicing. Small nuclear ribonucleoproteins (snRNPs) recognize the 5′ splice donor (GU) and the 3′ splice acceptor (AG) flanking adjacent exons. Multiple ribonucleoprotein components assemble into the spliceosome complex, which excises introns and ligates exons to form mature mRNA.

Splicing is a post-transcriptional RNA processing mechanism widely conserved among eukaryotes, in which non-coding introns are excised and coding exons are ligated, generally for translation into functional proteins. Across eukaryotes, splicing occurs in multiple forms, ranging from canonical constitutive and alternative splicing to diverse non-canonical mechanisms (Figure 1, Table 1). Under normal circumstances, the spliceosome recognizes a canonical 5′splice donor, typically a GU dinucleotide at the 5′end of the intron, and a canonical 3′splice acceptor, typically an AG dinucleotide at the 3′end, to define each splicing reaction. Both canonical splice sites and the mediation of the spliceosome are essential components of canonical splicing. Canonical splicing is sometimes completed through a strict adherence to a fixed exon-intron structure, yielding a single, obligatory mature transcript; this process is termed constitutive splicing. Conversely, through alternative combinations of exons within a single gene, known as alternative splicing, this process generates multiple mRNA isoforms that expand proteomic diversity and contribute to gene regulation (Modrek and Lee, 2002; McManus and Graveley, 2011; Kelemen et al., 2013; Marasco and Kornblihtt, 2023) (Figure 2).

**TABLE 1:** Comparison of the canonical and non-canonical splicing types discussed in this review, together with their principal characteristics.

Class	Characteristics
Canonical constitutive splicing	All exons within a single pre-mRNA are joined sequentially to produce a single transcript isoform
Canonical alternative splicing	Exons within a single pre-mRNA are joined in a selective manner, with one or more exons potentially skipped, thereby generating multiple transcript isoforms
Non-canonical splice sites	One or both nucleotides at the splice junction deviate from the canonical GT-AG consensus, resulting in splice sites that do not conform to the standard motif
Non-canonical lncRNA splicing	Commonly involves splicing at non-canonical splice sites, most notably GC-AG splice junctions
Microexon splicing	Splicing involving extremely short exons, typically only 3–15 nucleotides in length
Recursive splicing	Splicing of exceptionally long introns that contain multiple internal splice sites in addition to their terminal boundaries, allowing intron removal through a stepwise series of successive splicing reactions
Back splicing	A splicing event in which a downstream splice acceptor is joined to an upstream splice donor, often giving rise to circular RNAs (circRNAs)
Trans-splicing	A splicing process in which a transcript is generated by joining sequences derived from multiple pre-mRNAs of the same gene, different genes, or even different chromosomes
tRNA splicing	An essential step in tRNA maturation, mediated by a set of enzymes distinct from the spliceosome
IRE1-mediated splicing	A spliceosome-independent form of cytoplasmic splicing mediated by IRE1, occurring only in specific target transcripts such as XBP1 or HAC1

In eukaryotes, pre-mRNA splicing is carried out by two related ribonucleoprotein machineries: the major (U2-type) spliceosome, which mediates more than 99% of splicing events, and the minor (U12-type) spliceosome, which accounts for less than 1% (Lin et al., 2010; Kretova et al., 2023). The U2-type spliceosome is typically composed of the small nuclear ribonucleoproteins (snRNPs) U1, U2, U4, U5, and U6, whereas the U12-type spliceosome generally consists of U11, U12, U4atac, U5, and U6atac (Bradley and Anczukow, 2023; Kretova et al., 2023). The intron classes processed by these two spliceosomes differ in the conservation and architecture of their recognition signals. U2-type introns generally exhibit less constrained splice-site consensus sequences, which contributes to greater flexibility in splice-site selection and facilitates alternative splicing; they also typically contain a more prominent 3′ polypyrimidine tract (PPT, also known as Py-tract). In contrast, U12-type introns are characterized by more highly conserved recognition elements. Their 3′-end recognition depends more strongly on a highly conserved branch point sequence, and they often lack the canonical 3′ PPT typical of U2-type introns (Wu and Fu, 2015; Akinyi and Frilander, 2021). With respect to terminal splice-site dinucleotides, canonical U2-type splicing predominantly follows the GT–AG rule, whereas U12-type splicing is most associated with the AT–AC motif, although a small subset of U12-type introns also uses GT–AG motif (Turunen et al., 2013; El Marabti et al., 2021).

A canonical splicing event typically follows these principles: introns and exons of sufficient lengths, major canonical donor and acceptor sites (GT–AG) contributed sequentially by the preceding exon and the following exon, and mediation by the spliceosome. However, exceptions to these canonical rules have been increasingly documented. Collectively termed non-canonical splicing, these events do not strictly follow this paradigm. Some remain partially recognized and processed by the spliceosome, whereas others proceed through distinct mechanisms and yield diverse molecular outcomes.

## 2 Splicing with non-canonical splice sites

While canonical splicing relies on well-defined splice site signals and a conserved exon–intron architecture, accumulating evidence indicates that splice site recognition is not an all-or-nothing process. Instead, the spliceosome exhibits a measurable degree of tolerance to deviations from the major canonical GT–AG consensus, giving rise to splicing events that operate at the boundary of canonical rules. These deviations are most prominently manifested at the level of splice site sequences themselves and represent one of the most fundamental forms of non-canonical splicing. Accordingly, an examination of non-canonical splice sites provides a critical entry point for understanding how canonical splicing principles are relaxed, modified, or repurposed to achieve regulatory flexibility.

**FIGURE 3:**
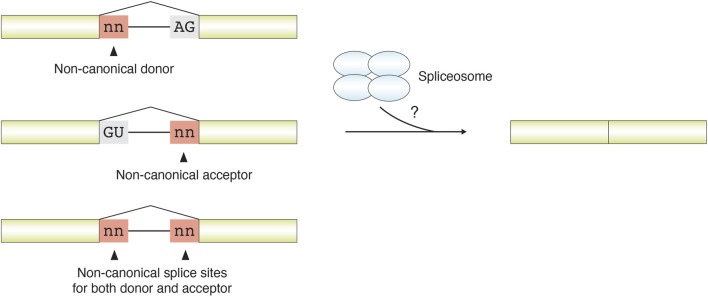
Schematic illustration of splicing with non-canonical splice sites. Variations in splice sites can be categorized into events involving a non-canonical splice donor only, a non-canonical splice acceptor only, or deviations at both sites. Among these events, some are still tolerated and mediated by the U2/U12 spliceosome, whereas others appear to proceed through diverse and gene-specific mechanisms that remain to be further elucidated.

Splicing events involving non-canonical splice sites do not conform to the canonical GT–AG motif. Depending on the context, such events may involve a non-canonical donor site, a non-canonical acceptor site, or deviations at both ends (Figure 3). Although splice-site recognition by the spliceosome is dominated by canonical GT–AG signals, multiple lines of evidence indicate that spliceosomal processing is not strictly limited to GT–AG junctions. A subset of non-canonical splice sites can still be recognized and excised when their surrounding sequence context retains features compatible with U2- or U12-type splice-site recognition (Turunen et al., 2013; Parada et al., 2014; Akinyi and Frilander, 2021). In such cases, the terminal dinucleotides deviate from the canonical pattern, but the broader splice-site architecture remains sufficiently similar to known major or minor spliceosomal recognition signals. In addition, some non-canonical splice sites may become functionally more canonical-like through context-dependent mechanisms such as A-to-I RNA editing or may show evolutionary relationships to nearby or ancestral canonical splice sites (Parada et al., 2014). Non-canonical junctions that retain recognizable U2/U12-like consensus features are often described as U2/U12-like non-canonical junctions. By contrast, non-U2/U12-like non-canonical junctions are not readily explained by established major or minor spliceosomal recognition models and may represent a heterogeneous class that includes both mechanistically distinct RNA-processing events and lower-confidence events influenced by technical artifacts (Parada et al., 2014; Sibley et al., 2016).

Non-canonical splice sites are exceedingly rare among all splicing events. In a previous systematic baseline study, splice sites outside the canonical GT–AG class accounted for only about 1% of all events (Parada et al., 2014). Moreover, when the major U2/U12-like non-canonical classes, namely GC–AG and AT–AC, are excluded, the proportion of all remaining minor non-canonical splice-site combinations decreases further to only 0.08% (Parada et al., 2014). Similarly, another study focused on non-canonical 5′ splice sites found that non-canonical 5′ splice junctions represented only 0.4%–1% of all exon junctions (Erkelenz et al., 2018). Taken together, these observations indicate that splicing involving non-canonical splice sites constitutes a minority class rather than a mainstream mode of splicing.

For such rare splicing events, methodological differences can substantially affect prevalence estimates. For example, in the earlier systematic baseline study, after functional relevance filtering, the number of retained non-canonical splice sites was reduced to 28% of the broader set identified by library-guided detection (462 of 1,630), and after further classification into U2/U12-like sites, this number dropped even more sharply, to only 11% of the initially detected candidate set (184 of 1,630) (Parada et al., 2014). Likewise, in the study of non-canonical 5′ splice sites, the raw RNA-seq estimate placed non-canonical 5′ splice junctions at 2.2%, whereas removal of low-usage junctions likely to represent noise reduced the high-confidence proportion to only 0.4%–1% (Erkelenz et al., 2018). These findings show that differences in filtering stringency and functional criteria can materially alter the reported prevalence of non-canonical splice sites across studies; however, even when considered together, published estimates consistently place them within a very small fraction of total splicing events. Moreover, because splicing itself is inherently heterogeneous and dynamic, caution is warranted when applying functionality-based filters to non-canonical splice sites, as overly stringent or overly permissive thresholds may distort their apparent prevalence.

With respect to sequencing modality, the overall distribution of non-canonical splice sites inferred from long-read sequencing has not shown major divergence from estimates derived from short-read sequencing. Nevertheless, the large number of novel splice junctions uncovered by long-read approaches remains an important subject for further investigation. A genome-wide survey of non-canonical splice sites in plants reported that, under long-read sequencing, all non-canonical splice sites together accounted for approximately 1.35%, whereas minor non-canonical splice sites represented only 0.09% (Pucker and Brockington, 2018). In addition, a long-read transcriptomic characterization of mouse neural tissue reported that only 0.1% of all known splice junctions were non-canonical (Tardaguila et al., 2018), and a study of rainbow trout genome annotation likewise reported approximately 0.1% non-canonical junctions (Ali et al., 2021). Collectively, these long-read-based studies are broadly consistent with the view that non-canonical splicing represents a very low-abundance class of splicing events.

At the same time, long-read sequencing has revealed a large number of previously undetected and uncharacterized splice sites, typically classified as novel splice junctions, which appear to have remained relatively underexplored. Many studies report these novel splice junctions as a separate category in long-read RNA-seq analyses; however, because the primary focus of those studies often lies elsewhere, these junctions are not always subjected to further filtering, remapping, or mechanistic analysis (Wright et al., 2022; Humphrey et al., 2025). Determining to what extent such novel junctions correspond to *bona fide* non-canonical splicing events therefore represents an important emerging challenge. In this regard, the earlier long-read transcriptomic study of mouse neural tissue is particularly informative, as it further analyzed novel splice junctions and found that 31% were classified as non-canonical junctions (Tardaguila et al., 2018). This suggests that long-read sequencing can uncover a substantial number of novel isoforms and potentially informative splice features that may be missed, or at least insufficiently resolved, by short-read approaches. However, an important issue has also emerged: novel-calling in long-read data appears to be highly sensitive to pipeline design and artifact control (Tardaguila et al., 2018; Su et al., 2024), indicating that further improvements in quality-control procedures are still needed. Moreover, there is currently a lack of dedicated long-read-based baseline surveys of non-canonical splice sites, making it difficult to determine whether the large number of novel isoforms identified by long-read sequencing substantially alters the baseline frequencies previously inferred from short-read data.

Non-canonical splice sites can also display tissue-specific expression patterns. In terms of gene regulation, they are often surrounded by an increased density of splicing regulatory elements (SREs), indicating that more complex regulatory architectures may be required for their recognition (Parada et al., 2014; Vuong et al., 2016; Yang et al., 2023). Moreover, certain non-canonical splice sites themselves contribute to gene regulation; for example, some can trigger nonsense-mediated mRNA decay (NMD). In the mouse brain, specific 3′splice sites within the *Syngap1* gene, which encodes the Ras GTPase-activating protein SYNGAP1, have been shown to induce NMD, leading to mRNA degradation and haploinsufficiency of SYNGAP1 protein and thereby affecting neuronal function and behavior (Yang et al., 2023). Coincidentally, additional non-canonical splice sites and truncated exon variants have also been reported in *Syngap1* (Kilinc et al., 2022). Building on these observations, we re-analyzed RNA-seq data from mouse neuronal cells described in the above studies (Kilinc et al., 2022; Yang et al., 2023) to further characterize the prevalence and sequence features of non-canonical splice sites.

### 2.1 Sequence enrichment of non-canonical splice sites

A sequence-enrichment analysis was performed on RNA-seq reads from EGFP+ cells sorted from E14.5 Tubb3-EGFP transgenic mice (Yang et al., 2023). Among all detected splice junctions, canonical GT–AG junctions were the most abundant, accounting for approximately 98.9% of the total (Table 2). These were followed by the major U2/U12 spliceosomal non-canonical preference sites, GC–AG and AT–AC, which accounted for approximately 0.83% and 0.12%, respectively, with the remaining fraction comprising other minor non-canonical splice sites (Table 2). The most prevalent splice-site classes and their relative proportions were highly similar to those reported in previous baseline studies (Parada et al., 2014).

**TABLE 2:** Distribution of splice-site dinucleotide combinations among all detected splice junctions. Each splice-site class is defined by the donor and acceptor terminal dinucleotides. The table reports the absolute count and relative frequency (%) of each splice-site combination across all detected junctions.

Splice site	Count	%	Splice site	Count	%	Splice site	Count	%
GTAG	149,786	98.9438	GCCC	2	0.0013	CCGT	1	0.0007
GCAG	1,253	0.8277	GCGG	2	0.0013	GCGA	1	0.0007
ATAC	185	0.1222	ACCG	1	0.0007	TGTT	1	0.0007
GTTG	33	0.0218	AGTG	1	0.0007	GGAA	1	0.0007
GTGG	21	0.0139	CTAG	1	0.0007	GGTC	1	0.0007
ATAG	11	0.0073	GTTC	1	0.0007	GGCC	1	0.0007
GAAG	9	0.0059	TGGA	1	0.0007	CTCC	1	0.0007
TTAG	8	0.0053	TCAG	1	0.0007	TGTC	1	0.0007
ATAA	8	0.0053	ACAC	1	0.0007	TTTT	1	0.0007
ATAT	6	0.0040	GCCT	1	0.0007	AGGG	1	0.0007
GGAG	6	0.0040	AGCC	1	0.0007	GGGT	1	0.0007
GTAA	4	0.0026	TTCT	1	0.0007	CTAT	1	0.0007
GGAC	3	0.0020	GGGC	1	0.0007	CTAA	1	0.0007
GTCA	3	0.0020	CCTT	1	0.0007	CAAC	1	0.0007
TGTG	2	0.0013	TCCC	1	0.0007	TCTT	1	0.0007
TATC	2	0.0013	CCCC	1	0.0007	AAGG	1	0.0007
GGGA	2	0.0013	CCGG	1	0.0007	TCGG	1	0.0007
AGAG	2	0.0013	CTGA	1	0.0007	GACA	1	0.0007
TAGA	2	0.0013	GATG	1	0.0007			

**FIGURE 4:**
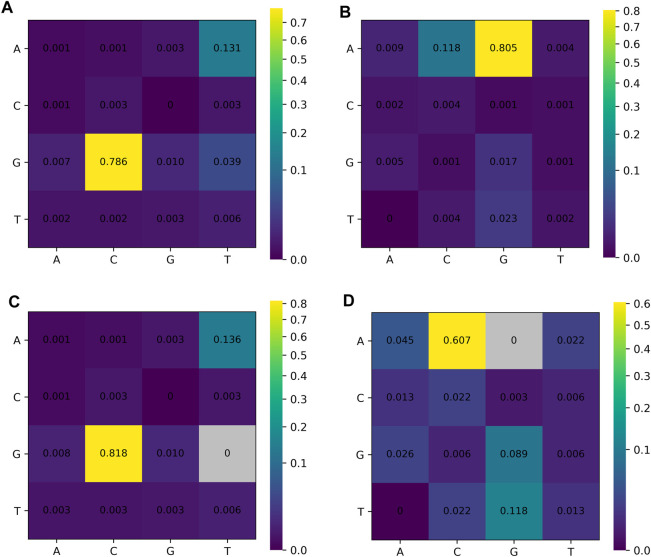
4 × 4 dinucleotide heat-map analysis of non-canonical splice sites: **(A)** donors, **(B)** acceptors, **(C)** donors with GT excluded, and **(D)** acceptors with AG excluded. All non-canonical splice sites were first counted and then converted to frequencies. Rows correspond to the first nucleotide of the splice site and columns to the second. Heat-map colors were scaled using power-law normalization (PowerNorm, gamma = 0.5) to enhance the visual separation of low-frequency minor non-canonical dinucleotide classes. This normalization was applied only to the color scale while the underlying counts/frequencies and the numeric labels remained untransformed.

Examination of the 5′- and 3′-terminal dinucleotides of all non-canonical introns revealed that AG remained by far the most frequently used non-canonical acceptor. Although GT was less frequent among non-canonical donor sites than GC and AT, which were observed mainly in the U2/U12-like GC–AG and AT–AC junctions, GT still occurred more frequently than most other minor donor classes (Figures 4A,B). In other words, GT donor sites and AG acceptor sites remained representative among non-canonical splicing events, which means a proportion of the non-canonical junctions were only non-canonical at one end and retained a canonical dinucleotide at the opposite splice site. These findings further indicate that many mapped non-canonical splice sites still retain at least one canonical donor or acceptor dinucleotide, strongly suggesting that the spliceosome can accommodate a certain degree of non-canonical variation at one or both ends. Although the major U2/U12-like non-canonical classes, GC–AG and AT–AC, accounted for a large fraction of this category, it should also be recognized that this group still includes a subset of single-sided non-canonical splice sites (Table 2), whose underlying splicing mechanisms are also expected to be compatible with spliceosomal recognition. Among splice sites lacking the GT–AG motif, GC and AT emerged as two frequent alternative donor dinucleotides, whereas AC was the most common alternative acceptor (Figures 4C,D). This enrichment is also likely attributable to the frequent occurrence of GC–AG and AT–AC junctions. In addition, TG and GG were also observed at relatively high frequencies as alternative acceptors (Figures 4C,D). Notably, the GG acceptor displayed potential functional links to the non-canonical splicing events reported in SYNGAP1 and to the activation of nonsense-mediated mRNA decay (NMD) (Kilinc et al., 2022; Yang et al., 2023), highlighting a promising direction for future investigation.

### 2.2 Difference in intron length between canonical and non-canonical introns

**FIGURE 5:**
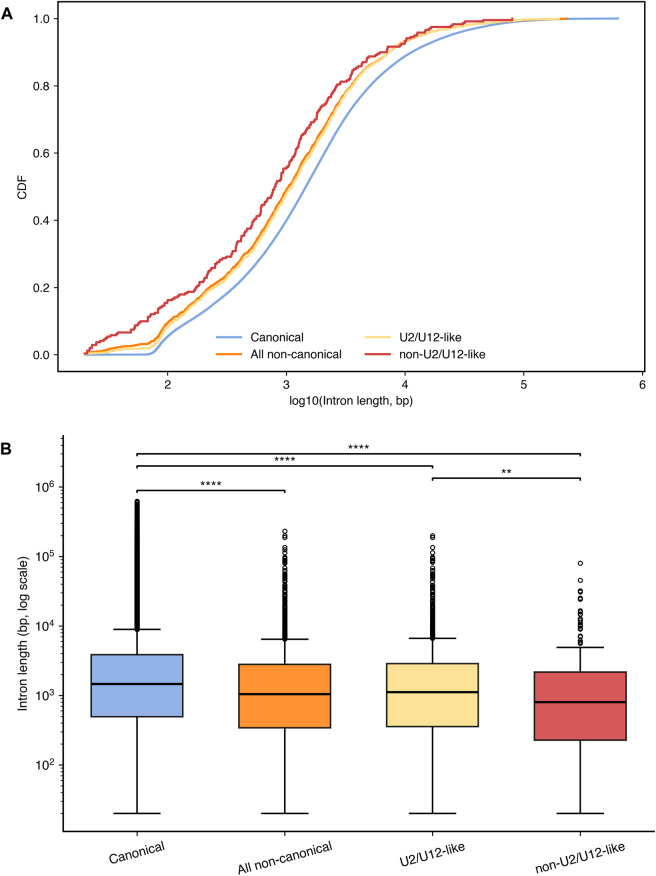
**(A)** Empirical cumulative distribution function (ECDF) plot and **(B)** box plot showing differences in intron lengths (log10-scaled) between canonical and non-canonical introns (Mann–Whitney U test, p = 3.657 × 10^−18^), canonical and non-canonical U2/U12-like introns (p = 2.904 × 10^−12^), canonical and non-canonical non-U2/U12-like introns (p = 1.494 × 10^−9^), and non-canonical U2/U12-like and non-U2/U12 like (p = 2.544 × 10^−3^). Individual points shown in the boxplots represent outlier observations only, defined as values lying beyond 1.5 × the interquartile range from the box, rather than all underlying data points.

Within the same sample, introns excised at non-canonical splice sites were significantly shorter than canonical introns, with a median length difference of 415.5 bp (Figure 5; Mann–Whitney U test, p = 3.657 × 10^−18^). After further stratification of all non-canonical splice introns into U2/U12-like non-canonical and non-U2/U12-like non-canonical subsets, both groups likewise showed significantly shorter introns than canonical introns. Specifically, the median intron length of the U2/U12-like non-canonical subset was 343 bp shorter than that of canonical introns (Figure 5; p = 2.904 × 10^−12^), whereas the median intron length of the non-U2/U12-like non-canonical subset was 659.5 bp shorter (Figure 5; p = 1.494 × 10^−9^). These findings strongly suggest that, regardless of whether a non-canonical junction is still likely to be processed by the U2/U12 spliceosome, its associated intron tends to be shorter than those of canonical splice sites. Interestingly, introns in the non-U2/U12-like non-canonical subset were also significantly shorter than those in the U2/U12-like non-canonical subset (Figure 5; p = 2.544 × 10^−3^). These results indicate that non-U2/U12-like non-canonical introns exhibit a more pronounced shortening relative to canonical introns than do U2/U12-like non-canonical introns, which may reflect underlying differences in their splice-site recognition mechanisms, including possible differences in sequence conservation. Accordingly, a tendency toward shorter introns may serve as a useful indicator in future studies of non-canonical splicing for further identifying and prioritizing non-U2/U12-like splicing events.

### 2.3 Future perspectives of splicing with non-canonical splice sites

Our analysis indicates that a substantial proportion of non-canonical splicing events recognized in mouse neuronal cells involve splicing with only a single non-canonical splice site, remarkably for the acceptor. This suggests that many non-canonical splicing events remain mediated by the U2/U12 spliceosome. At the same time, beyond the major U2/U12-preferred non-canonical junction classes, such as GC–AG and AT–AC, some junctions with a splice-site change at only one end are also likely to be tolerated and successfully processed by the U2/U12 spliceosome. In addition, introns involved in splicing with non-canonical splice sites, especially non-U2/U12-like non-canonical splice sites, are significantly shorter than those associated with canonical splicing events. Given the persistent challenges in accurately identifying diverse forms of non-canonical splicing, a bias toward shorter intron length may serve as an auxiliary criterion for the detection and characterization of non-canonical splicing events. Moreover, as non-canonical splice sites in SYNGAP1 mRNA can potentially trigger NMD, leading to reduced dosage or altered forms of proteins essential for neurodevelopment, the role of aberrant splicing in the pathogenesis of various diseases warrants further investigation.

The continued development of long-read sequencing has revealed many previously unclassified novel splicing isoforms, which are likely to contain substantial numbers of as-yet undiscovered, unclassified, and uncharacterized non-canonical splice sites. Accordingly, systematic investigation of non-canonical splice sites in the context of long-read sequencing, together with the establishment of comprehensive baseline surveys comparable to those developed in the short-read era, represents an important and timely direction for future research.

While the above features outline the general landscape of non-canonical splice sites across transcripts, their distribution and functional relevance are not uniform across RNA classes. Notably, long non-coding RNAs (lncRNAs) exhibit a distinct enrichment of non-canonical splice sites (Khan et al., 2023), providing a permissive context in which spliceosomal tolerance may be amplified and repurposed for regulatory functions.

## 3 Non-canonical splicing in long non-coding RNAs (lncRNAs)

**FIGURE 6:**
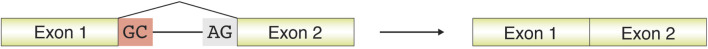
A representative and relatively conserved form of non-canonical splicing in lncRNAs. This scenario exemplifies splicing with a non-canonical donor only, in which a non-canonical dinucleotide (GC) replaces the canonical GU at the 5′ splice donor of the first exon in an lncRNA transcript.

Compared with protein-coding genes, lncRNAs display a markedly higher frequency of non-canonical splice sites, reflecting reduced coding constraints and increased splicing plasticity (Figure 6). lncRNAs are RNA transcripts longer than 200 nt but lack the potential to be translated into proteins (Mattick et al., 2023). lncRNAs also undergo splicing, and the vast majority are mediated by the spliceosome. However, compared to mRNA, lncRNA exhibits distinct differences in selective pressure, functional constraints, and splicing precision (Derrien et al., 2012; Chen and Kim, 2024). This renders lncRNA a domain where non-canonical splicing occurs more readily yet proves more challenging to define.

### 3.1 Instability of lncRNA splicing mechanisms

Compared with mRNA splicing, lncRNA splicing is generally characterized by weaker splice site strength, lower conservation of exon-intron architecture, and a markedly higher prevalence of alternative splicing, with intron retention and exon skipping representing common features rather than exceptions in lncRNAs (Ouyang et al., 2022; Khan et al., 2023). Notably, alternative splicing of lncRNAs is particularly prevalent and can regulate selective splicing through multiple mechanisms, including interactions with splicing factors, base pairing with pre-mRNAs, and modulation of chromatin remodeling (Pisignano and Ladomery, 2021; Ouyang et al., 2022). Such variability and instability in splicing provide a broad landscape for the emergence and diversification of non-canonical splicing events.

### 3.2 Non-canonical splice sites in lncRNAs

The presence of weak splice sites and the high splicing plasticity characteristic of lncRNAs provide a structural basis for non-canonical splicing, particularly for the emergence and utilization of non-canonical splice sites. The occurrence of non-canonical splice sites is markedly higher in lncRNAs than in protein-coding genes (PCGs) (Khan et al., 2023). The GC–AG pattern represents a non-canonical splicing mode that is markedly enriched in lncRNAs. In both human and mouse lncRNAs, GC–AG motifs occur at frequencies approximately two-to four-fold higher than those observed in protein-coding genes and are preferentially enriched within the first intron (Abou Alezz et al., 2020). In splicing events involving non-canonical sites, both donor and acceptor splice sites are significantly weaker, and the associated polypyrimidine tracts are shorter (Abou Alezz et al., 2020; Statello et al., 2021). Such structural features render these sites more susceptible to further alternative splicing and show a strong association with alternative polyadenylation, collectively indicating that non-canonical splice sites play a regulatory role in lncRNA splicing (Abou Alezz et al., 2020).

Non-canonical splicing in lncRNAs remains a relatively infrequent class of RNA-splicing events overall, yet its reported prevalence is consistently higher than that observed in protein-coding genes (PCGs), with no major disagreement across published studies. A previous review summarized that atypical splice sites account for approximately ∼3% of splice sites in lncRNAs, compared with ∼0.8% in PCGs (Khan et al., 2023). Another study similarly reported that non-GT–AG introns comprise approximately 3.4% of lncRNA splice junctions in humans, compared with 1.4% in PCGs; in mice, the corresponding values were 3.4% and 1.2%, respectively (Abou Alezz et al., 2020). Together, these studies support a consistent overall conclusion: although non-canonical splicing in lncRNAs remains a minority phenomenon, its usage is higher than in protein-coding genes.

### 3.3 Future perspectives of non-canonical splicing in lncRNAs

lncRNAs undergo pervasive alternative splicing, frequently generating aberrant isoforms implicated in oncogenesis (Ouyang et al., 2022; Wang et al., 2022). The extensive splicing variability of lncRNAs, together with their documented roles in tumorigenesis, demonstrates that lncRNAs are far from mere transcriptional noise but instead represent a long-overlooked class of regulatory molecules with substantial functional significance in gene regulation. Beyond the already highly diverse landscape of canonical alternative splicing, lncRNAs that undergo non-canonical splicing also warrant close attention, as they may uncover conserved splicing mechanisms or serve as indicators of aberrant splicing events that contribute to disease pathogenesis.

## 4 Splicing with microexons

While non-canonical splice sites challenge the sequence constraints of spliceosome recognition, other non-canonical splicing events arise even when canonical splice signals are preserved. Microexons exemplify such cases, in which extreme exon length rather than splice site identity imposes unique regulatory and kinetic constraints on splicing.

**FIGURE 7:**
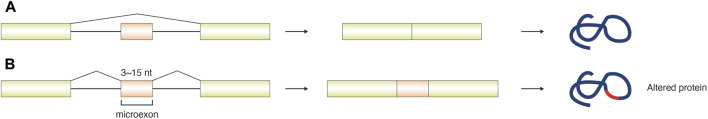
Schematic illustration of splicing involving microexons. This process can also occur in a regulated, alternative manner: **(A)** the microexon is skipped and removed as part of the intron; **(B)** the microexon is recognized as an exon and incorporated into the mature transcript. When retained in mRNA transcripts, microexons can give rise to distinct protein variants upon translation.

Microexons are very short exonic sequences, typically only 3–15 nucleotides in length, with tightly regulated splicing and high evolutionary conservation (Li et al., 2015; Mackensen and Irimia, 2025) (Figure 7). Once largely overlooked, they are now recognized as modulators of diverse protein functions and interactions, particularly in the central nervous system (Irimia et al., 2014; Li et al., 2015; Mackensen and Irimia, 2025). Microexons show a marked tendency for in-frame insertion, which can fine-tune protein properties (Irimia et al., 2014; Li et al., 2015). Mechanistically, microexons are similar to canonical exons, but their unusually short length has long impeded their reliable detection by standard RNA-seq alignment and transcriptome annotation methods.

### 4.1 Microexons are often tightly regulated

The inclusion of microexons in mature mRNA is stringently regulated across tissues and developmental stages. RNA-seq analyses have shown that, in both humans and mice, the vast majority of microexons are detected in transcripts associated with the central nervous system, whereas they are seldom found in non-neural tissues (Irimia et al., 2014; Li et al., 2015). These observations indicate that microexon splicing is highly tissue-specific and closely linked to neural development and function.

### 4.2 Many microexons exhibit strong evolutionary conservation

Microexons display highly similar sequences, splicing structures, and regulatory patterns across species. Cross-species analyses have revealed nearly identical exon sequences and flanking splice sites in numerous neural microexons, and the patterns of percent spliced in (PSI) likewise show similar inclusion trends between mammals (Irimia et al., 2014; Ustianenko et al., 2017). Most microexons are integer multiples of three nucleotides—typically 3 to 15 bases, and occasionally up to 27 or even 51 bases—allowing their inclusion without disrupting the coding frame (Li et al., 2015). Remarkably, even the shortest microexons, containing only 3–6 nucleotides, display highly conserved patterns that are rarely observed in typical exons (Li et al., 2015).

### 4.3 Alterations and functions of microexons on mRNAs and proteins

Through in-frame splicing, microexons can modify the amino acid sequence without compromising the overall structural integrity of the encoded protein. The microexon-encoded residues are typically inserted into intrinsically disordered regions (IDRs)—flexible linkers between structured domains—or into loops on domain surfaces, thereby fine-tuning protein interactions and dynamics. These additional amino acids are frequently charged residues, such as glutamate (E), aspartate (D), lysine (K), or arginine (R) (Dergai et al., 2010; Irimia et al., 2014; Li et al., 2015).

### 4.4 Relevance of microexons to neural tissues and neurological disorders

Microexons—short exons of typically 3–15 nucleotides—exhibit a strong neural tissue–specific expression pattern and participate in diverse neuronal processes, including neuronal maturation, synaptic plasticity, cytoskeletal remodeling, and signal integration (Irimia et al., 2014; Quesnel-Vallières et al., 2016). Dysregulation of neuronal microexon splicing has been reported in the brains of individuals with autism spectrum disorder (ASD) (Irimia et al., 2014; Quesnel-Vallières et al., 2016), suggesting that modulating microexon inclusion could provide novel avenues for the treatment of neurodevelopmental or neurodegenerative disorders.

### 4.5 Discussion about prevalence of microexon splicing

Microexon splicing has likewise been widely reported as a relatively rare class of splicing event; however, despite substantial differences in study design and statistical definitions, microexons are also consistently described as having a high degree of involvement in neural-regulated alternative splicing (AS). Microexon-type AS events account for only about 1% of all AS events (Irimia et al., 2014; Li et al., 2015). In a study of autistic brains, 60.7% of alternative microexons showed increased neural PSI, whereas only 9.5% of longer alternative exons did so, indicating a marked enrichment of microexons in neural function–related splicing programs (Irimia et al., 2014). Interestingly, in another study of micro-exons in the human brain that did not explicitly report a comparable neural-PSI-based probability, the proportion of brain-expressed microexons among all detected microexons was also 60.7% (7,949 of 13,095) (Li et al., 2015). However, this numerical agreement is likely coincidental rather than biologically equivalent, because the two studies addressed different questions: the former quantified neural inclusion at the level of alternative splicing, whereas the latter measured only expression in brain-associated transcripts. This distinction implies that the actual proportion of microexons showing preferential inclusion in the brain in the latter study was likely lower than 60.7%.

Such differences may reflect variation in tissue scope and dataset scale (mouse and human vs. human only), RNA-seq processing strategies (a multi-module exon–exon junction library approach vs. Augmented Transcriptome Mapping), and microexon filtering criteria (3–15 nt vs. ≤51 nt, with candidates shorter than 6 nt discarded). Taken together, these observations suggest that microexons have not yet been studied within a unified baseline framework; instead, they have often been filtered and analyzed according to disease-specific or regulatory priorities. Combined with the inherent technical difficulty of detecting microexons, this has left the field without a broadly accepted and widely used standard analytical framework, and it therefore remains comparatively underexplored. Given that the continued development of long-read sequencing is enabling the detection of increasing numbers of microexons (Rahimi et al., 2021a; 2021b), long-read-based studies of microexons, particularly in the context of neural regulation, represent a promising direction for future research.

### 4.6 Future perspectives of microexon splicing

Due to their extremely short length and consequent difficulty in detection, microexons are systematically underestimated by current short-read RNA-seq approaches, and their annotation remains broadly incomplete. Furthermore, because many studies of RNA splicing remove very short introns and exons during filtering by treating them as noise, many potential microexons may be discarded at an early stage and consequently never enter downstream analyses. Advances in long-read sequencing, specialized alignment algorithms, and single-cell full-length RNA-seq are expected to substantially enhance the identification and characterization of microexons (Fuentes-Beals et al., 2023; Liu Z. et al., 2023; Fu et al., 2025). Meanwhile, the regulatory mechanisms governing microexon splicing remain incompletely understood. For example, how the spliceosome reliably recognizes highly conserved microexons, and why microexons are selectively included under specific cellular contexts, are still open questions. Given that microexon inclusion can generate distinct protein variants and that microexons are evolutionarily conserved in the nervous system, elucidating the mechanisms underlying microexon splicing may provide novel insights into diverse neuronal processes as well as neurodegenerative diseases.

## 5 Recursive splicing

**FIGURE 8:**
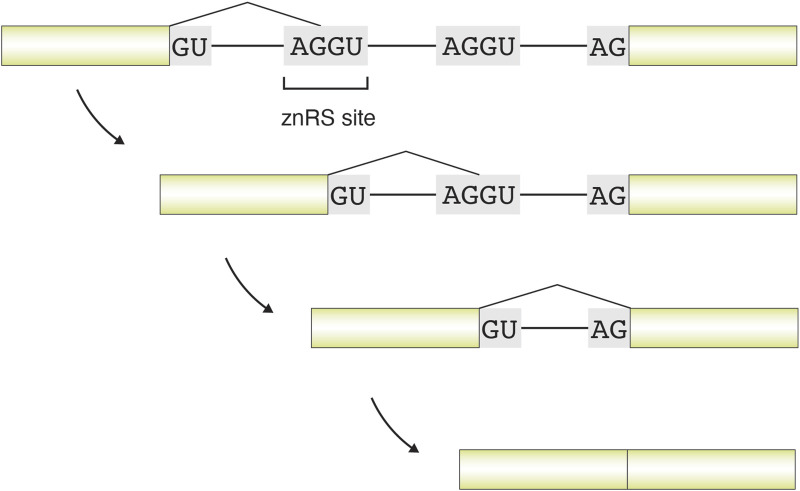
Schematic illustration of recursive splicing involving three consecutive splicing steps. In this example, a long intron contains two zero-nucleotide recursive splice (znRS) sites. In the first splicing step, the 5′ donor contributed by the upstream exon is joined to the AG acceptor of the first znRS site. In the second step, the remaining GU of the first znRS site functions as a donor and is spliced to the AG acceptor of the second znRS site. In the third step, the remaining GU of the second znRS site serves as the donor and is joined to the 3′ acceptor contributed by the downstream exon. This schematic depicts recursive splicing as a temporally ordered, kinetic splicing process.

Beyond exon definition, the spliceosome also faces challenges imposed by intron length. In genes containing exceptionally long introns, recursive splicing provides a stepwise strategy to maintain splicing fidelity and efficiency. Recursive splicing (RS) is a stepwise RNA splicing mechanism that segments the removal of very long introns—typically tens to hundreds of kilobases—into multiple successive steps (Figure 8). This strategy enhances splicing efficiency and reduces the risk of mis-splicing or the formation of aberrant isoforms. It proceeds through embedded recursive splice sites (RS sites) within the intron, which act as internal splice junctions and allow the intron to be excised fragment by fragment.

### 5.1 Structure and function of recursive splice sites

Recursive splicing typically relies on multiple AG|GU motifs embedded within long introns, which serve as relay points during the stepwise removal of a single long intron. These consecutive splice signals, in which a 3′AG acceptor is immediately followed by a 5′GU donor, are known as zero-nucleotide recursive splice sites (znRS sites), reflecting the absence of intervening nucleotides. In the first splicing step, a znRS site functions as a relay 3′acceptor, pairing with the original 5′donor to excise the initial intronic segment. The same znRS element then acts as a 5′donor in the next step, joining with the downstream AG acceptor, and this cycle can repeat when multiple znRS sites are present. Ultimately, znRS elements are either removed together with the intron (RS-excluded) or retained as optional exonic segments (RS-included), potentially generating alternative protein isoforms (Burnette et al., 2005; Duff et al., 2015; Sibley et al., 2015; Georgomanolis et al., 2016; Hayashi et al., 2018). The stepwise reuse of recursive splice sites inherently links recursive splicing to splicing dynamics and kinetics, as each splicing event creates a transient intermediate that temporally defines subsequent splice site recognition (Liu M. et al., 2023; Merens et al., 2024).

### 5.2 The distribution of recursive splicing

Recursive splicing was first identified in specific *Drosophila* genes and corresponding mRNA isoforms (Hatton et al., 1998), and has since been documented in mice and humans, with more than 100 RS sites reported (Duff et al., 2015; Sibley et al., 2015). These sites are typically located in the middle of long introns exceeding 10 kb in length. Moreover, recursive splicing occurs frequently in genes involved in nervous-system function, developmental regulation, and signal transduction (Duff et al., 2015; Sibley et al., 2015). Its high prevalence in the nervous system likely reflects the greater intron length and the need for complex splicing programs that generate multiple isoforms. This stepwise splicing mechanism is thought to enhance overall splicing efficiency, reduce mis-splicing events and transcriptional backlogs, and coordinate with co-transcriptional processing in these gene contexts.

Because recursive splicing (RS) remains a comparatively underexplored field, there is currently no baseline-level estimate of its overall prevalence. Conservatively, RS appears to be extremely rare based on the evidence currently available, and its prevalence may plausibly be even lower than that of non-canonical splice sites or microexons (i.e., below 1%), although the possibility of substantial underestimation cannot be excluded. In one study of RS in long vertebrate genes, approximately 1.04% of a broad candidate set of unique, unannotated splice sites (419 of 40,163) matched the RS-site motif, yet only 9 high-confidence RS-sites remained after further filtering, suggesting that RS may indeed be highly uncommon (Sibley et al., 2015). At the same time, contemporaneous and subsequent studies have still not been able to establish a definitive estimate of the overall prevalence of RS (Duff et al., 2015; Moon and Zhao, 2022; Hoppe et al., 2023).

What can be stated more confidently at present is that RS is strongly enriched in long introns. A study using *Drosophila* as a genetic model reported that approximately 70% of long *Drosophila* introns (>40 kb) contain at least one RS-site (Kumari et al., 2022). Another genome-wide study in *Drosophila* further showed that 97% of all introns are smaller than the smallest recursive intron, providing additional indirect support for a close association between RS and very long introns (Duff et al., 2015). However, one review of RS has pointed out that previous searches for RS relied too heavily on very long introns as a defining criterion, potentially restricting the detectable scope of RS events (Hoppe et al., 2023). Given the uncertainty surrounding its overall prevalence, together with possible biases in search scope, RS remains a field with substantial room for further investigation.

### 5.3 Future perspectives of recursive splicing

Although recursive splicing has gained increasing recognition as an efficient strategy for processing long introns, its overall prevalence and regulatory mechanisms remain incompletely understood. Given that recursive splicing is a dynamic, multi-step process, many events are likely to evade detection by conventional short-read sequencing approaches. Long-read and nascent RNA sequencing may therefore reveal a substantially larger and more dynamic landscape of recursive splicing (Wan et al., 2021; Liu M. et al., 2023). From an evolutionary perspective, the reasons why long introns and recursive splicing are conserved in nervous-system–associated genes warrant further investigation, and such insights may provide new entry points for the study of neurodegenerative diseases. Mechanistically, the multi-step and dynamic nature of recursive splicing suggest the presence of temporal and context-dependent regulation. How the spliceosome selectively recognizes functional recursive splice sites within long introns, and how this selection is coordinated with transcriptional dynamics, remain open questions.

## 6 Back splicing and circular RNAs (circRNAs)

While recursive splicing extends splicing across time, other non-canonical events fundamentally alter the topology of exon ligation. Back-splicing represents such a topological departure, in which splice sites are joined in a reverse orientation to generate circular RNAs.

**FIGURE 9:**
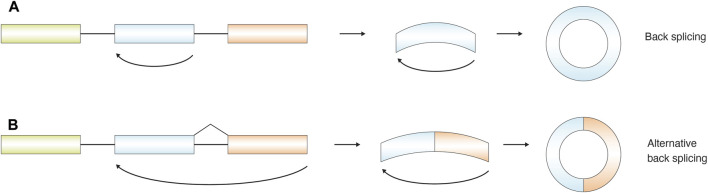
Schematic illustration of the back-splicing process and the formation of circular RNAs (circRNAs). Back-splicing can occur **(A)**within a single exon or **(B)**selectively between multiple exons, resulting in the generation of distinct RNA lariats. In multi-exon back-splicing events, intronic sequences are sometimes retained, giving rise to circRNAs that contain intronic regions.

Back splicing is a special form of RNA splicing in which a downstream 5′splice donor site of an exon is joined to an upstream 3′splice acceptor, rather than to the 3′acceptor of a downstream exon as in canonical splicing. This atypical ligation generates a covalently closed circular RNA (circRNA) (Figure 9A). In some cases, back-splicing can occur across multiple exons. Canonical splicing events still take place internally, while the most upstream splice donor is joined to the most downstream splice acceptor, resulting in alternative back-splicing and the formation of circular RNAs containing multiple exons (Zhang et al., 2016; Chen, 2020) (Figure 9B).

### 6.1 Mechanism of back splicing and circRNA biogenesis

A covalently closed circular RNA (circRNA) is generated when a downstream 5′splice donor joins an upstream 3′splice acceptor—opposite to the direction of canonical (linear) splicing. Back-splicing can be mechanistically categorized into three principal models: intron-pairing–driven circularization, RNA-binding-protein (RBP)–mediated circularization, and lariat-driven circularization (Kristensen et al., 2019). In the intron-pairing–driven model, complementary Alu elements—short interspersed nuclear elements (SINEs) within flanking introns—form base-paired secondary structures that bring the 5′and 3′splice sites into proximity, thereby promoting exon back-splicing (Jeck et al., 2013). In the RBP-mediated model, specific RBPs recognize cis-acting motifs in upstream and downstream introns and bridge them to form a loop, facilitating circRNA formation (Ashwal-Fluss et al., 2014; Conn et al., 2015). The lariat-driven model involves the coexistence of exon skipping and back splicing: a spliced lariat containing skipped exons undergoes internal splicing to yield a circRNA (Zhang et al., 2013; 2016; Kelly et al., 2015). Notably, circRNA formation itself often undergoes alternative splicing, producing multiple circRNAs from a single gene through the use of alternative 5′or 3′back-splice sites, a process largely governed by competition among complementary intronic sequences (Zhang et al., 2016). Moreover, intronic regions can also circularize, generating circular intronic RNAs (ciRNAs) that lack exonic sequences (Zhang et al., 2013).

### 6.2 Molecular structures and translational potential of circRNAs

Owing to their covalently closed, head-to-tail circular structure, circRNAs lack free 5′and 3′ends and canonical splice junctions, making them largely resistant to exonucleolytic degradation and thereby substantially more stable than their linear RNA counterparts (Jeck et al., 2013; Enuka et al., 2016; Kristensen et al., 2019). Moreover, because circRNAs lack both a 5′cap and a 3′poly(A) tail, they are unable to initiate cap-dependent translation (Jeck et al., 2013; Enuka et al., 2016; Kristensen et al., 2019). Nevertheless, certain circRNAs have been shown to undergo translation via alternative mechanisms. For example, some harbor internal ribosome entry sites (IRES) that directly recruit ribosomal subunits in a cap-independent manner (Legnini et al., 2017; Yang et al., 2017). Others rely on an epitranscriptomic trigger in which N6-methyladenosine (m6A) modification recruits eIF3 and ribosomes to initiate translation independently of the 5′cap (Pamudurti et al., 2017; Yang et al., 2017; Zhou et al., 2019).

### 6.3 circRNAs as microRNA regulators

For a long time, circRNAs were primarily viewed as miRNA decoys or sponges that modulate miRNA-mediated regulation (Hansen et al., 2013). In parallel, numerous studies have attempted to engineer synthetic circRNAs targeting specific miRNAs, with the aim of exploiting their regulatory potential for the treatment of miRNA-associated diseases, including hepatitis C, gastric cancer, and type 2 diabetes (Rossbach, 2019; Avendaño-Portugal et al., 2025). However, it has gradually become apparent that circRNA-miRNA interactions are not limited to a simple sponge model. For example, CDR1as (CDR1 antisense) circRNA was initially proposed to function as a miR-7 sponge (Hansen et al., 2013; Memczak et al., 2013; Chen, 2020), but was later found to protect miR-7 from degradation mediated by the long noncoding RNA Cyrano (Kleaveland et al., 2018). This interplay forms a highly conserved regulatory network in the mammalian brain, where CDR1as modulates miR-7 availability and downstream target repression (Kleaveland et al., 2018). Loss of CDR1as leads to downregulation of miR-7 targets and to behavioral changes in mice, highlighting the physiological relevance of circRNA–miRNA interactions. In addition, emerging evidence suggests that the relationship between neuronal circRNAs and miRNAs may be better characterized by coordinated localization and local regulatory interactions, rather than by straightforward sequestration (Woods and Van Vactor, 2021; Silenzi et al., 2024).

Taken together, these findings suggest that circRNAs may influence miRNA regulation through a broader range of mechanisms beyond sponge activity. At the same time, a purely sponge-based regulation depends on several stringent parameters, including expression level, number of binding sites, binding affinity, subcellular localization, and stoichiometric relationship (Nielsen et al., 2022). Because such conditions are unlikely to be broadly met under physiological circumstances (Thomson and Dinger, 2016; Nielsen et al., 2022), this further supports the view that many circRNA regulators may affect miRNA function through mechanisms other than simple sponging.

### 6.4 Discussion about back splicing and circRNAs

Back-splicing appears to be more prevalent than several of the other non-canonical splicing events discussed above, and this field is correspondingly more mature. Nevertheless, as research progresses and sequencing technologies continue to improve, additional conserved back-splicing events and circRNAs are still being identified, and their tissue-specific expression patterns warrant further investigation. Long-read sequencing analyses have indicated that 4.44% of reads in the human brain and 6.23% of reads in the mouse brain contain circRNA back-splice junctions (Rahimi et al., 2021b). Other studies have identified more than 880,000 back-splicing events in humans, potentially giving rise to more than 1.8 million human circRNA isoforms (Margvelani et al., 2025). Together, these observations indicate that back-splicing and circRNAs are widespread in mammals. Additional studies have further shown that circRNAs are present at broadly comparable orders of magnitude in the brain, pituitary, ovary, and liver, suggesting that circRNA expression is pervasive across diverse tissues (Li et al., 2021); however, their tissue-specific and stage-specific expression patterns still require close attention.

Another important issue concerns the ongoing debate over the biological significance of back-splicing and circRNAs. CircRNAs have been widely reported to possess diverse biological functions, including the translational potential and microRNA-regulatory roles discussed above (Legnini et al., 2017; Pamudurti et al., 2017; Kleaveland et al., 2018), and their functions in regulation and coding have been broadly validated (Shi et al., 2023; Nemeth et al., 2024). At the same time, other studies have argued that mammalian circRNAs are produced primarily as by-products of splicing errors, and that as many as 97% of such events are deleterious (Xu and Zhang, 2021). One possible interpretation is that these two views are not necessarily contradictory. Rather, circRNAs with specific biological functions or conserved regulatory roles may represent only a minority, whereas the majority of circRNAs produced *in vivo* may indeed arise from erroneous back-splicing events. Accordingly, determining whether a circRNA is generated incidentally or is recurrently expressed within a given biological system may become one of the key criteria for distinguishing functional circRNAs from aberrant splicing products.

### 6.5 Future perspectives of back splicing and circRNAs

Benefiting from their exceptional resistance to exonucleolytic degradation, circRNAs hold promise as therapeutic agents, vaccine platforms, and gene-delivery vectors (Wesselhoeft et al., 2018; O’Leary et al., 2025). Engineered circRNAs exhibit prolonged half-lives and can support robust, sustained protein expression (Wesselhoeft et al., 2018; Chen, 2020). They have also been explored as vaccine platforms, including for SARS-CoV-2, where circRNA vaccines show improved stability compared to conventional mRNA vaccines (Qu et al., 2022; Niu et al., 2023). In cancer therapy, circRNAs can function as gene-therapy vectors, persisting and acting as gene medicines (Wesselhoeft et al., 2018). Moreover, shared molecular features of circRNAs in tumor cells provide a rationale for their use as cancer biomarkers, further broadening their diagnostic potential (Vo et al., 2019; Wang et al., 2021; Zhang et al., 2021). In addition, the regulatory effects of circRNAs on miRNAs, particularly those involving regulatory networks beyond simple sponging, remain an area with considerable potential for further investigation (Kleaveland et al., 2018; Woods and Van Vactor, 2021; Silenzi et al., 2024). Long-read sequencing has also provided critical evidence for the detection of circRNAs and the elucidation of back-splicing mechanisms, thereby compensating for the limitations of second-generation sequencing in identifying these non-canonical splicing events (Rahimi et al., 2021a). Back-splicing and circRNAs exhibit substantial potential in RNA regulation, such as through microRNA sponge activity, as well as in disease prevention and treatment, including applications as circRNA-based vaccines and cancer biomarkers.

## 7 Trans-splicing

Whereas back-splicing reshapes exon connectivity within a single transcript, trans-splicing further expands splicing plasticity by linking exons derived from distinct pre-mRNA molecules, sometimes even across genes or chromosomes.

**FIGURE 10:**
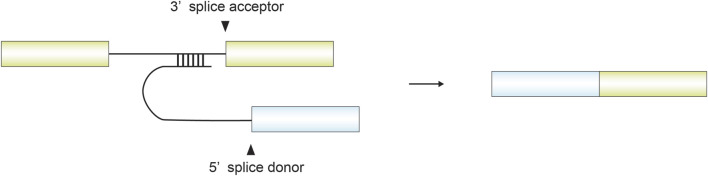
Schematic illustration of trans-splicing between two distinct genes. Intronic sequences from one gene form complementary interactions with intronic regions of another gene, bringing the flanking exons into proximity and enabling their ligation. Each exon from the two genes contributes either a splice donor or a splice acceptor, resulting in the formation of a chimeric transcript.

Trans-splicing is a form of non-canonical splicing in which exons derived from separate pre-mRNA precursors are joined to generate a single mature mRNA (Figure 10). Unlike canonical cis-like splicing, which ligates exons within a single transcript, trans-splicing can link exons originating from different pre-mRNAs of the same gene, from distinct genes, or even from different chromosomes, thereby expanding the diversity of mRNA transcripts and protein isoforms.

### 7.1 Structure and pattern of trans-splice sites

Trans-splicing follows the canonical GT–AG splice motif even though it joins exons derived from two different pre-mRNA molecules (Horiuchi and Aigaki, 2006). These splice junctions can be intragenic, arising from different promoters within the same gene, or intergenic, connecting exons from different genes or even different chromosomes (Horiuchi and Aigaki, 2006). The *mod (mdg4)* gene in *Drosophila* exemplifies an intragenic trans-splicing event: exon 5 is transcribed in the opposite direction to exons 1-4 and is joined to them through trans-splicing (Büchner et al., 2000; Dorn et al., 2001; Dorn and Krauss, 2003; McManus et al., 2010). At the other extreme, the human *ACAT1* gene provides an example of interchromosomal trans-splicing, in which a functional chimeric mRNA is formed from exons located on chromosome 7 and chromosome 1 (Li et al., 1999). This interchromosomal splicing has been shown to generate proteins with defined biological functions (Chen et al., 2008). Although all of these events share the canonical GT–AG splice motif, their underlying mechanisms vary, with some cases relying on RNA–RNA base pairing—such as complementary arm sequences—to bring distant transcripts into physical proximity (Dorn et al., 2001; Dorn and Krauss, 2003).

### 7.2 Functions of trans-splicing

Trans-splicing events between distinct pre-mRNAs directly promote the production of additional mRNA transcripts and protein isoforms, thereby diversifying gene expression and regulatory networks (Gingeras, 2009). In *Drosophila*, trans-splicing of the mod (mdg4) gene contributes more than 30 protein isoforms that influence chromatin organization and regulation (Büchner et al., 2000; Dorn and Krauss, 2003). In certain contexts, trans-splicing can also trigger interallelic complementation, in which homologous recombination between two alleles regenerates a functional mRNA transcript. This process can mediate gene repair or functional restoration (Horiuchi et al., 2003).

### 7.3 The prevalence of trans-splicing in *Caenorhabditis elegans* (*C. elegans*) is remarkably high

Early transcriptome-wide surveys estimated that roughly 70%–85% of *C. elegans* genes undergo trans-splicing, predominantly mediated by SL1 or SL2 leader sequences (Allen et al., 2011; Ramani et al., 2011). More recent long-read RNA-seq analyses have confirmed and extended these findings, showing that about 87.4% of genes are trans-spliced (Bernard et al., 2023), indicating that transcript processing in *C. elegans* follows a mechanism distinct from that of other eukaryotes.

### 7.4 Discussion about trans-splicing

The prevalence of trans-splicing is strongly dependent on both species and tissue context. For example, in the mitochondrial genomes of Pinaceae and Cupressaceae plants, where exons are dispersed, approximately 50%–70% of mitochondrial introns require trans-splicing (Guo et al., 2020). In *C. elegans*, 87.4% of genes undergo trans-splicing (Bernard et al., 2023). By contrast, in humans, trans-splicing is generally considered a rare event, and a substantial fraction of human trans-spliced chimeric RNAs appears to arise from chromosomal rearrangements in cancer cells (Yokomori et al., 2024). For instance, in one analytical set compiled from previously published human trans-spliced genes, only 52 genes were classified as trans-spliced, compared with 24,808 non-trans-spliced genes (Yokomori et al., 2024). In addition, one integrative transcriptome study reported that, among 24,498 detected non-co-linear splicing events, approximately 20%–40% represented mixtures of trans-spliced RNAs and circRNA isoforms (Chuang et al., 2018), further suggesting a potential connection between trans-splicing and circRNA biogenesis.

Although a subset of experimentally observed trans-splicing events has been confirmed to be functional, many are likely to represent biological noise or technical byproducts. Studies have shown that numerous reported cases may arise as artifacts during cDNA preparation or sequencing, in which reverse transcriptase and related enzymes can induce template switching when encountering direct repeat sequences, thereby generating chimeric reads that mimic trans-splicing events (Cocquet et al., 2006; Houseley and Tollervey, 2010; Kong et al., 2015; Balázs et al., 2019). Consequently, careful experimental validation and stringent bioinformatic filtering are essential to distinguish *bona fide* trans-splicing from artifacts and background noise.

Taken together, trans-splicing can exhibit strikingly different patterns of enrichment across species, tissues, and even individual genes. This indicates that current research has already achieved a certain breadth in characterizing trans-splicing, but its relationships with chromosomal rearrangements, circRNAs, and disease warrant much deeper investigation. In addition, background noise and false-positive signals remain important concerns that must be carefully addressed in studies of trans-splicing.

### 7.5 Future perspectives of trans-splicing

Although false positives and background noise remain major challenges in the study of trans-splicing, emerging technologies and bioinformatic tools have begun to dispel some of the uncertainty surrounding this field (Liu et al., 2020). Meanwhile, attempts to integrate trans-splicing with CRISPR–Cas platforms to develop novel RNA-editing tools have also been reported (Fiflis et al., 2024; Zhu et al., 2024; Chandrasekaran et al., 2026). Continued development and application of these approaches will facilitate a more comprehensive understanding of trans-splicing. At the functional level, trans-splicing directly expands transcript and protein diversity and holds research potential in chromatin regulation, transcript complementation, and gene repair. *C. elegans*, in which trans-splicing operates as a predominant mechanism of transcript processing, offers a valuable evolutionary framework for exploring the origins and significance of trans-splicing. Moreover, as a shared question across many spliceosome-mediated non-canonical splicing events, the mechanisms by which the spliceosome tolerates trans-splicing between different genes or even across chromosomes remain to be elucidated.

## 8 Spliceosome-independent splicing

Collectively, the above non-canonical events illustrate the remarkable tolerance and adaptability of the spliceosome. However, not all RNA splicing reactions rely on spliceosomal machinery. In certain biological contexts, splicing is executed through entirely distinct enzymatic pathways.

Spliceosome-independent RNA splicing represents a mechanistically distinct pathway from canonical, spliceosome-mediated mRNA splicing. Two well-characterized examples include the endonuclease-mediated excision of introns from precursor tRNAs in eukaryotes and archaea, and the non-canonical cytoplasmic splicing mediated by the ER stress sensor IRE1 during unfolded protein response.

### 8.1 tRNA splicing

**FIGURE 11:**
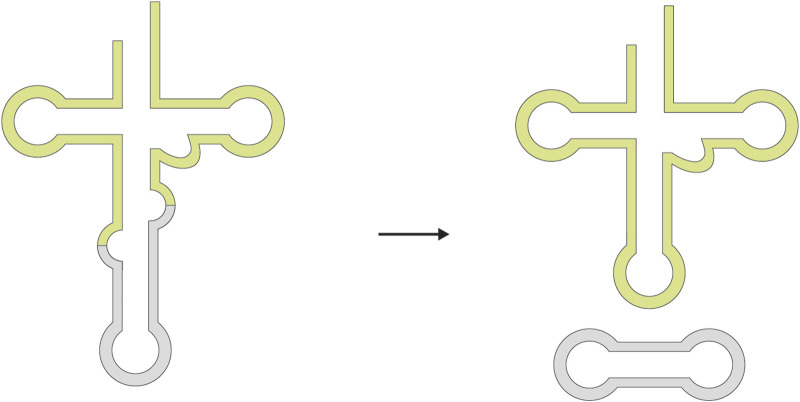
Simplified schematic of tRNA splicing. The intron within a precursor tRNA is excised by a site-specific endonuclease, generating an RNA lariat intermediate, after which the exons are ligated by a tRNA ligase. This process is carried out independently of the spliceosome.

tRNA splicing does not rely on the spliceosome, the ribonucleoprotein machinery used for canonical mRNA splicing but instead depends directly on specialized protein enzymes (Figure 11). Consequently, its reaction pathway differs substantially from canonical splicing. In eukaryotes, each pre-tRNA is first cleaved by a site-specific endonuclease, yielding two tRNA exon halves and a linear intron (Abelson et al., 1998). The exon halves are then ligated by tRNA ligase, producing a full-length tRNA bearing a 2′-phosphate at the splice junction, and this residual phosphate is subsequently removed by cyclic phosphodiesterase (CPDase) to yield a functional tRNA (Abelson et al., 1998). In archaea, pre-tRNAs contain a bulge-helix-bulge (BHB) structural motif that is precisely recognized and cleaved by the endonuclease (Belfort and Weiner, 1997; Trotta et al., 1997). Both eukaryotic and archaeal systems thus rely on spatial constraints and RNA secondary structure—rather than the GT–AG sequence motifs of canonical splicing—for accurate intron removal (Reinhold-Hurek and Shub, 1992; Abelson et al., 1998; Fujishima and Kanai, 2014). By contrast, enzyme-dependent tRNA splicing is rare in bacteria, where most introns are excised through self-splicing mediated by the tRNA’s own structure (Belfort and Weiner, 1997; Trotta et al., 1997; Abelson et al., 1998).

tRNA splicing is an obligate maturation step during pre-translation preparations, and is therefore widely observed in eukaryotes and archaea, although its prevalence varies across lineages and its underlying mechanisms differ substantially. In contrast, it is not a general feature of bacterial tRNA maturation. Although tRNA splicing has been widely studied, ongoing discoveries at the mechanistic and structural levels continue to expand our understanding of this process. Certain enzymes have been identified that do not participate directly in tRNA splicing itself but instead modify tRNAs during post-splicing maturation (Hayne et al., 2022). During tRNA splicing, a circular RNA can also be generated, and the potential regulatory roles of this non-coding lariat in RNA regulation warrant further investigation. Moreover, several enzymes involved in tRNA splicing have been implicated in integrated stress responses and certain neurological disorders (Hurtig and van Hoof, 2023; Phizicky and Hopper, 2023), suggesting that further studies may provide important insights into the treatment of these diseases.

### 8.2 IRE1-mediated cytoplasmic splicing

**FIGURE 12:**
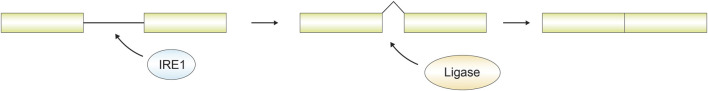
Simplified schematic of IRE1-mediated splicing. The intron is first excised by the endoribonuclease IRE1, after which a specific RNA ligase joins the two exons. The coordinated action of IRE1 and the ligase functionally substitutes for the spliceosome in this splicing process.

Inositol-requiring enzyme 1 (IRE1) is an endoplasmic reticulum transmembrane sensor that is responsible for maintaining endoplasmic reticulum homeostasis and overall cellular function (Siwecka et al., 2021; Le Goupil et al., 2024). In certain contexts, IRE1 can function analogously to the spliceosome by mediating the splicing of specific mRNAs in the cytoplasm (Figure 12). This process is spliceosome-independent and, unlike canonical splicing, does not occur in the nucleus.

A representative example is the cytoplasmic splicing of X-box binding protein 1 (XBP1) mRNA in metazoans. The splicing of XBP1, a core transcription factor in the mammalian endoplasmic reticulum (ER) stress response, occurs in the cytoplasm and is mediated by the endoribonuclease IRE1 (Yoshida et al., 2001; Lee et al., 2002; Back et al., 2006; Uemura et al., 2009; Yanagitani et al., 2011). Under ER stress, IRE1 cleaves XBP1 pre-mRNA at two conserved RNA stem–loop structures that serve as splice sites, and an RNA ligase then joins the exons, allowing XBP1 to become functional and drive the transcription of molecular chaperones and ER-associated degradation (ERAD) components (Yoshida et al., 2001; Lee et al., 2002; Back et al., 2006; Uemura et al., 2009; Yanagitani et al., 2011). This cytoplasmic cleavage enables a rapid restoration of ER homeostasis. Notably, XBP1 splicing bypasses the canonical GU–AG motif and does not generate a lariat intermediate; instead, the intron is directly removed by IRE1 (Yoshida et al., 2001; Lee et al., 2002; Back et al., 2006; Uemura et al., 2009; Yanagitani et al., 2011).

Another example of IRE1-mediated cytoplasmic splicing is the splicing of Homologous to ATF/CREB 1 (HAC1) in *Saccharomyces cerevisiae*. HAC1 is the functional homolog of XBP1 in yeast, and its splicing likewise occurs in the cytoplasm, with the intron being directly cleaved by IRE1 and the exons subsequently ligated by an RNA ligase (Xia, 2019). In higher plants, the bZIP60 mRNA likewise undergoes IRE1-mediated cytoplasmic splicing in response to ER stress (Kaur and Kaitheri Kandoth, 2021; Li et al., 2025).

Compared with the non-canonical splicing events discussed above, in which the spliceosome remains involved, IRE1-mediated splicing is target-restricted rather than globally prevalent and has been reported only in a very small number of distinct regulatory systems. IRE1 can cleave the transcripts described above to maintain endoplasmic reticulum homeostasis and support cell survival. Conversely, IRE1 can also target ER-associated RNAs for cleavage and subsequent degradation, a process that may, in some contexts, lead to cell death (Tam et al., 2014; Bartoszewska et al., 2023). How IRE1 discriminates between transcripts destined for productive splicing and those targeted for degradation remains an important open question. This dual role of IRE1 as a “double-edged sword” in ER signaling underscores its central regulatory importance and highlights its potential as a therapeutic target for drug development.

## 9 Conclusions and future directions

In this review, we have summarized and discussed multiple forms of non-canonical alternative splicing, outlining their principal structures, current landscapes, challenges, and prospective directions for future research (Table 3).

**TABLE 3:** Comparative summary of the major forms of non-canonical splicing discussed in this review, highlighting their current research landscape, key challenges, and future directions.

Class	Current landscape	Challenges	Possible future directions
Non-canonical splice sites	Long-read sequencing has revealed large numbers of novel, uncharacterized junctions Reported to be associated with NMD and neurological disease	Prevalence estimates are highly sensitive to analytical stringency Long-read novel-calling is pipeline- and artifact-sensitive Non-U2/U12-like junctions remain mechanistically heterogeneous and poorly resolved	Build long-read baseline surveys comparable to short-read-era studies Test disease relevance and tissue specificity, especially in neural and NMD-linked contexts Further investigate the different mechanisms of non-U2/U12-like non-canonical splicing
Non-canonical lncRNA splicing	Non-canonical splice site usage is more prevalent than in PCGs GC–AG introns are enriched, especially in first introns Weak splice signals may underlie high splicing plasticity	Mechanistic basis of lncRNA-specific non-canonical splicing remains unclear Current evidence remains limited compared with PCGs	Define the regulatory roles of non-canonical splicing in lncRNAs Clarify disease relevance, especially in cancer-associated lncRNA isoforms
Microexon splicing	Long-read sequencing is revealing additional microexons Specialized pipelines are improving microexon detection Single-cell full-length RNA-seq is enabling context-specific analysis	Reported prevalence is difficult to compare across studies because the underlying definitions differ Short-read RNA-seq and standard filtering likely underestimate microexons	Develop long-read-based and specialized pipelines for microexon detection Clarify how the spliceosome recognizes extremely short microexons Define the roles of microexons in neural regulation and neurological disease
Recursive splicing	Strongly enriched in very long introns Reported to be enriched in neuronal cells Recent studies suggest that earlier searches may have underestimated its scope	Overall prevalence remains poorly defined Detection is limited by short-read sequencing and search-scope bias Mechanistic regulation and site selection remain incompletely understood	Use long-read and nascent RNA sequencing to detect dynamic RS events Clarify how functional RS-sites are recognized within long introns Determine how RS is coordinated with transcriptional dynamics and neural gene regulation
Back splicing and circRNAs	Long-read sequencing is expanding the detectable landscape Recognized as widespread across multiple mammalian tissues; circRNAs are increasingly explored for RNA vaccines and biomarker applications	Distinction between functional circRNAs and aberrant by-products remains unresolved Most circRNAs may not be functionally equivalent despite widespread detection Tissue-specific and stage-specific expression patterns remain incompletely characterized	Use long-read sequencing to improve detection and analysis Develop criteria to distinguish recurrent functional circRNAs from incidental products Expand therapeutic and biomarker studies of circRNAs in disease contexts Explore broader miRNA-regulatory roles of circRNAs beyond conventional sponge activity
Trans-splicing	Emerging sequencing and bioinformatic tools are improving detection Beginning to be explored in CRISPR-based RNA-editing platforms	False positives and background noise remain major concerns Mechanistic links to chromosomal rearrangements, circRNAs, and disease remain incompletely resolved	Develop more stringent validation frameworks for *bona fide* trans-splicing Expand RNA-editing and gene-repair applications based on trans-splicing
tRNA splicing	Widely conserved in eukaryotes and archaea Reported to be implicated in integrated stress responses and certain neurological disorders	Functions of certain involving enzymes remain unclear Connection to specific stress responses and disorders remains unclear	Define how tRNA-splicing factors intersect with stress-response pathways Investigate the mechanistic links between tRNA splicing and neurological disease
IRE1-mediated splicing	A representative example of spliceosome-independent splicing Restricted to a small number of key ER stress-responsive transcripts Conserved across distinct systems	Additional forms of spliceosome-independent splicing are likely to remain unidentified How IRE1 distinguishes productive splicing from RNA decay remains unclear	Explore broader landscape of similar spliceosome-independent cases Clarify how IRE1 selects transcripts for splicing versus degradation Develop therapeutic strategies targeting IRE1-dependent stress signaling

Non-canonical splicing comprises a broad and mechanistically diverse set of RNA-processing events. Accordingly, not all forms of non-canonical splicing should be discussed at the same analytical level. A useful organizing principle is to classify these events according to their degree of deviation from spliceosome-dependent splicing. For example, many cases of splicing with non-canonical splice sites, particularly the U2/U12-like subset, can still be tolerated by the spliceosome and are best regarded as spliceosome-mediated. Events that depart from canonical splicing in certain mechanistic aspects but still remain partially compatible with spliceosomal recognition may be more appropriately grouped as spliceosome-compatible, including microexons, recursive splicing, and trans-splicing. By contrast, forms of non-canonical splicing that operate through fundamentally distinct mechanisms, such as tRNA splicing or the splicing of XBP1 and HAC1, are more appropriately classified as spliceosome-independent. These three categories differ substantially in mechanism and should therefore be analyzed separately. At the same time, this framework is not absolute. Some reported subclasses of non-canonical splicing may span conceptual boundaries; for instance, certain minor non-canonical splice junctions that do not conform to current U2/U12-like models may nonetheless rely on as-yet-uncharacterized mechanisms, potentially placing them closer to the spliceosome-compatible or even spliceosome-independent end of the spectrum. In such cases, individual events may require case-by-case discussion, as is already common for XBP1 and HAC1.

A second general conclusion is that reported prevalence is highly method dependent. Because published studies differ substantially in scope, period, and analytical design, the resulting prevalence estimates and reporting frameworks are often difficult to compare directly. Two issues are particularly important. First, the increasing use of long-read sequencing has not fundamentally overturned the overall view of non-canonical splicing, but it has revealed a large number of junctions that were not detectable in the short-read era, thereby exposing a substantial and still underexplored landscape. These newly detected long-read junctions represent an important resource for future investigation. Second, studies often differ markedly in pipeline stringency and filtering strategy, because different analytical aims naturally lead to different data-processing workflows and parameter choices. As a result, cross-study comparisons of non-canonical splicing require careful attention to methodology. When necessary, reanalysis of public raw datasets under a consistent filtering framework may provide a more reliable basis for direct comparison.

Species specificity and tissue specificity also emerge as important dimensions of non-canonical splicing. For example, trans-splicing is almost a dominant mode of transcript processing in *C. elegans*, whereas microexons, recursive splicing, and non-canonical splice sites have all been repeatedly linked to the nervous system in multiple studies. More broadly, these forms of potentially neural-associated non-canonical splicing may contribute to a deeper understanding of neuronal RNA processing and of neurological disease.

Finally, mechanistic stratification is likely to change interpretation. In the earlier RNA-seq reanalysis presented in this review, non-canonical introns were compared with canonical introns only as a single combined category. However, after further classification based on U2/U12-like scoring, a previously unanticipated conclusion emerged: non-canonical non-U2/U12-like introns also tended to be shorter than non-canonical U2/U12-like introns. Deeper subdivision and mechanistic categorization are essential for the study of non-canonical splicing. With the support of long-read sequencing, the field has already achieved considerable breadth; however, more refined mechanistic dissection at greater depth is likely to be especially valuable in the future.

## 10 Supplementary methods

### 10.1 RNA-seq mapping

Paired-end RNA-seq reads from EGFP+ cells sorted from E14.5 Tubb3-EGFP transgenic mice (NCBI Accession: SRR23308049) (Yang et al., 2023) were aligned to the mouse GRCm39 reference genome using STAR (v2.7.10b) (Dobin et al., 2013) with a GRCm39 genome index from NCBI RefSeq using --sjdbOverhang 59. Alignment was performed in single-pass mode with default splice-junction output filtering, and splice junctions were extracted from SJ.out.tab.

### 10.2 Data categorization and filtering

Using Python (v3.13.2), splice junctions in SJ.out.tab were first separated into canonical and non-canonical junctions and written to two corresponding output files. Only junctions supported by more than one uniquely mapped read (unique_reads > 1) were retained. Relevant features were preserved for downstream analysis, including chromosome, genomic start and end coordinates, strand, junction motif, gene name, and intron length.

Among all splice junctions identified by STAR, all junctions other than those with the GT–AG pattern (motif_code ≠ 1) were initially treated as non-canonical junctions and were subsequently subjected to further classification in downstream analyses based on their surrounding sequence features. This strategy was adopted because, in addition to GT–AG junctions, STAR also assigns several major classes of non-canonical junctions to predefined motif categories, for example motif_code = 2 for CT-AC and motif_code = 3 for GC-AG. Because the downstream analysis involved a more detailed PWM-based scoring framework for identifying U2/U12-like junctions, STAR’s built-in classification of these major non-canonical classes was not used for the final classification step.

In addition, because STAR does not directly assign gene names to non-canonical junctions, non-canonical splice sites were further remapped using the GRCm39 GTF annotation index. Putative gene assignment was inferred by determining whether each junction fell within the genomic span of an annotated gene.

All source code has been uploaded to GitHub (https://github.com/SpringShann/Non-canonical-RNA-splicing-tools-for-STAR), where the attached example inputs and outputs are taken from the original data of this study.

### 10.3 U2/U12-like categorization for non-canonical splice sites

Using canonical splice sites identified from the same sample, position weight matrices (PWMs) were constructed to assess whether non-canonical splice sites were consistent with U2/U12-like splicing. For canonical donor sites, a window comprising 3 nt upstream + the 2-nt donor site + 4 nt downstream (total length, 9 nt) was extracted. For canonical acceptor sites, a window comprising 18 nt upstream + the 2-nt acceptor site + 3 nt downstream (total length, 23 nt) was extracted. A longer window was used for acceptor sites because the natural sequence intervals typically associated with canonical donor and acceptor sites span approximately 10 bp and 28 bp, respectively (Caminsky et al., 2014). The trained PWMs were then used to score all non-canonical junctions, yielding a donor score, an acceptor score, and a total score for each junction. Scoring thresholds were determined using scrambled controls, in which randomized sequence windows were scored to generate a null score distribution. The threshold derived from this null distribution was then applied to the total score: junctions with total scores greater than the threshold were classified as U2/U12-like, whereas those with total scores less than or equal to the threshold were classified as non-U2/U12-like.

Non-canonical junctions were classified as not classified if no valid PWM score could be obtained, including cases in which strand orientation could not be resolved, sequence-window extraction failed, or the extracted window contained bases other than A, C, G, or T. These junctions were excluded from downstream statistical analyses.

The source code for this analysis is also available at GitHub (same depository).

### 10.4 4 × 4 dinucleotide heatmap analysis

Using Python (v3.13.2), the dinucleotide composition (i.e., motif patterns) of the non-canonical splice sites extracted in Section 10.2, Data categorization and filtering, was quantified. The results were visualized as separate 4 × 4 heatmaps for donor and acceptor sites. For each site class, dinucleotides were first counted as raw occurrences and then normalized to relative frequencies by dividing by the total number of valid sites. For better visualization of the minor non-canonical dinucleotides, color intensities were mapped using a power-law normalization (PowerNorm, gamma = 0.5) rather than a strictly linear scale. This transformation was applied only to the color mapping to improve the visual separation of low-frequency minor non-canonical dinucleotide classes when one or a few high-frequency classes dominated the distribution. The underlying count and frequency values, as well as the numeric annotations shown in each cell, were not transformed.

Because many non-canonical splicing events are non-canonical on only one side, with the opposite side still carrying a canonical dinucleotide, additional filtering was applied to remove GT from donor sites and AG from acceptor sites. Additional 4 × 4 heatmaps were then generated to visualize dinucleotide distributions after exclusion of these canonical site signals. In all heatmaps, rows correspond to the first nucleotide of the splice site and columns correspond to the second nucleotide.

The source code for this analysis and plotting is also available at GitHub (same depository).

### 10.5 Intron length comparison

Using Python (v3.13.2), intron lengths were extracted separately for canonical, non-canonical, non-canonical U2/U12-like, non-canonical non-U2/U12-like splice sites, respectively. The resulting distributions were visualized as (1) an empirical cumulative distribution function (ECDF) of intron lengths after log10 transformation, and (2) a boxplot of the raw intron lengths displayed on a log-scaled y-axis. In addition, a two-sided Mann–Whitney U test (Wilcoxon rank-sum test) was used to assess whether the distributions differed significantly. This nonparametric, rank-based test was selected because intron lengths are typically highly skewed and may span several orders of magnitude, making it more robust than parametric tests that assume approximate normality.

For the boxplot, outliers were defined according to the standard 1.5 × interquartile range rule and were displayed as individual points; all other observations were represented by the box, median line, and whiskers rather than plotted individually.

The source code for this analysis and plotting is also available at GitHub (same depository).
